# COVID-19 and Telepsychiatry: Development of Evidence-Based Guidance for Clinicians

**DOI:** 10.2196/21108

**Published:** 2020-08-28

**Authors:** Katharine Smith, Edoardo Ostinelli, Orla Macdonald, Andrea Cipriani

**Affiliations:** 1 Department of Psychiatry University of Oxford Oxford United Kingdom; 2 Oxford Health National Health Service Foundation Trust Oxford United Kingdom

**Keywords:** digital mental health, telepsychiatry, evidence-based guidance, systematic review, mental health, COVID-19

## Abstract

**Background:**

The coronavirus disease (COVID-19) presents unique challenges in health care, including mental health care provision. Telepsychiatry can provide an alternative to face-to-face assessment and can also be used creatively with other technologies to enhance care, but clinicians and patients may feel underconfident about embracing this new way of working.

**Objective:**

The aim of this paper is to produce an open-access, easy-to-consult, and reliable source of information and guidance about telepsychiatry and COVID-19 using an evidence-based approach.

**Methods:**

We systematically searched existing English language guidelines and websites for information on telepsychiatry in the context of COVID-19 up to and including May 2020. We used broad search criteria and included pre–COVID-19 guidelines and other digital mental health topics where relevant. We summarized the data we extracted as answers to specific clinical questions.

**Results:**

Findings from this study are presented as both a short practical checklist for clinicians and detailed textboxes with a full summary of all the guidelines. The summary textboxes are also available on an open-access webpage, which is regularly updated. These findings reflected the strong evidence base for the use of telepsychiatry and included guidelines for many of the common concerns expressed by clinicians about practical implementation, technology, information governance, and safety. Guidelines across countries differ significantly, with UK guidelines more conservative and focused on practical implementation and US guidelines more expansive and detailed. Guidelines on possible combinations with other digital technologies such as apps (eg, from the US Food and Drug Administration, the National Health Service Apps Library, and the National Institute for Health and Care Excellence) are less detailed. Several key areas were not represented. Although some special populations such as child and adolescent, and older adult, and cultural issues are specifically included, important populations such as learning disabilities, psychosis, personality disorder, and eating disorders, which may present particular challenges for telepsychiatry, are not. In addition, the initial consultation and follow-up sessions are not clearly distinguished. Finally, a hybrid model of care (combining telepsychiatry with other technologies and in-person care) is not explicitly covered by the existing guidelines.

**Conclusions:**

We produced a comprehensive synthesis of guidance answering a wide range of clinical questions in telepsychiatry. This meets the urgent need for practical information for both clinicians and health care organizations who are rapidly adapting to the pandemic and implementing remote consultation. It reflects variations across countries and can be used as a basis for organizational change in the short- and long-term. Providing easily accessible guidance is a first step but will need cultural change to implement as clinicians start to view telepsychiatry not just as a replacement but as a parallel and complementary form of delivering therapy with its own advantages and benefits as well as restrictions. A combination or hybrid approach can be the most successful approach in the new world of mental health post–COVID-19, and guidance will need to expand to encompass the use of telepsychiatry in conjunction with other in-person and digital technologies, and its use across all psychiatric disorders, not just those who are the first to access and engage with remote treatment.

## Introduction

The coronavirus disease (COVID-19) and the measures taken to limit its spread present unique challenges in all aspects of our everyday life. It is a rapidly progressing disease, evolving from its first description in December 2019 to a global pandemic with consequences worldwide [1]. In the absence of a new and effective vaccine, social distancing, isolation, and quarantine are the most effective interventions used across many countries to slow the spread of transmission [2].

These interventions have provided significant challenges for mental health care systems who (as part of wider health care) have been forced to reappraise ways of working in a rapid time frame, to change to telepsychiatry where possible and to provide adequate information technology systems for mental health care staff to provide remote care. This has happened in a time frame that in many countries, including the United Kingdom, would have been considered impossible only a few months ago [3]. In addition, the need for mental health support is likely to increase. Although COVID-19 is primarily a respiratory disease, data from the long-term neuropsychiatric sequelae of other severe coronavirus infections such as severe acute respiratory syndrome (SARS) and Middle East respiratory syndrome (MERS), and preliminary data for COVID-19 suggest not only significant rates of delirium in the acute stage but also depression, anxiety, fatigue, posttraumatic stress disorder (PTSD), and rarer neuropsychiatric syndromes in the longer term [4]. These longer-term mental health symptoms combined with the stresses of quarantine and self-isolation [5] are likely to increase demand for assessment and support from mental health services.

Despite evidence that e-therapy is equivalent to face-to-face therapy in terms of therapeutic alliance [6], there remains a concern from clinicians that telepsychiatry, in general, may not be as effective as “in-person” mental health care. In addition, clinicians are concerned that telepsychiatry cannot replace the team assessment and multidisciplinary approach that are at the center of modern mental health care provision [7]. It is also often assumed that patients may be reluctant or unable to engage with the technology involved. Mental health care provision, especially in the United Kingdom, has previously reflected this view [8], and although telepsychiatry has been introduced in the United Kingdom in some more remote areas [9], this has been, to at least some extent, out of necessity rather than preference.

In fact, the evidence, especially from the United States [10] shows the opposite. Telemedicine and telepsychiatry are well-established fields and are preferable in many areas such as for patients on the autistic spectrum and those with anxiety symptoms [11]. It can add value, for example, in bringing together subspecialty expertise in an easier and quicker way than in-person assessment, and in completing home and nursing home assessments more rapidly and efficiently. There is strong evidence of effectiveness and acceptability across different settings and many disciplines of psychiatry including older adult, child, and adolescent psychiatry, and across different cultures [11].

COVID-19 and its associated restrictions have prompted both clinicians and patients to reconsider telepsychiatry as a viable and valuable option. However, with change comes uncertainty and many clinicians in mental health feel unprepared for the new ways of working [12]. Areas of uncertainty such as issues of information governance, consent and confidentiality, accuracy of diagnosis, and modifications to any physical examination that may be appropriate are frequent questions in implementing digital technologies in the era of COVID-19 [8].

To integrate telepsychiatry successfully into the post–COVID-19 plan for clinical practice in psychiatry, clinicians, patients, and health care organizations need access to reliable and pragmatic clinical guidance and evidence [13]. In this rapidly evolving situation, with daily updates from specialties, countries, and world organizations, the amount of information available for the busy clinician can be overwhelming. We aim to meet this need by providing focused, evidence-based guidance on the use of digital technologies and telepsychiatry.

## Methods

A team of researchers with multidisciplinary backgrounds (including mental health clinicians, researchers, methodologists, and a pharmacist) from the Oxford Precision Psychiatry Lab [14] systematically searched English language websites from the United Kingdom, the United States, Australia, New Zealand, Canada, and Singapore for guidelines on telepsychiatry and telemedicine relevant to mental health and applicable in the context of the current COVID-19 pandemic and afterwards. We decided to focus on English language guidelines in the initial search both to meet the rapid time scale for focused guidance requested by our local clinicians and to include non-UK sites to ensure the synthesis of guidelines is relevant across other countries [13]. Two researchers (KS and EO) searched independently across the following sources in English, without age limits: Public Health England, Royal College of Psychiatrists, Royal College of Nursing, The National Association of Intensive Care and Low Secure Units, Royal College of Physicians, Healthcare Improvement Scotland, South London and Maudsley National Health Service (NHS) Trust, the National Institute for Health and Care Excellence, NHS Wales, General Medical Council, NHSX, Nursing and Midwifery Council (NMC), Centers for Disease Control and Prevention (CDC), US Department of Labor, American Psychiatric Association, Massachusetts General Hospital Department of Psychiatry, Federation of State Medical Boards (FSMB), Centers for Medicare and Medicaid Services (CMS), World Health Organization, Inter Agency Standing Committee, the United Nations International Children's Emergency Fund, World Psychiatric Association, Singapore Ministry of Health, Singapore Psychiatric Association, Singapore Medical Association, Health Canada, Canadian Psychiatric Association, Australian Government Department of Health, and the Royal Australian and New Zealand College of Psychiatrists. References to other sources from each website were also searched. Our search focused on guidelines for telepsychiatry in the context of COVID-19 up to and including May 2020. However, we decided to use a broad approach to the search to include both pre–COVID-19 guidelines where appropriate and guidelines for relevant digital technologies such as digital platforms for monitoring symptoms and the use of apps. A search on Google was also completed using keywords relevant to COVID-19 (eg, COVID-19, coronavirus, SARS-CoV-2 [severe acute respiratory syndrome coronavirus 2]), digital mental health (eg, digital mental health, telepsychiatry, digital psychiatry), and guidelines (eg, guideline, guidance, recommendation). Queries or disagreements were resolved by team discussion, and the team collaborated with an expert in the field to keep the guidance global, focused, and comprehensive.

The final synthesis of guidelines on telepsychiatry (including other relevant digital technologies) is presented here and is also available in an open-access webpage [15] in three different formats: (1) a webpage with embedded hyperlinks for online viewing, (2) a downloadable PDF for saving or printing, and (3) a detailed Word (Microsoft Corporation) document with sources and all information. Feedback and corrections from readers of the webpage are actively invited. Questions and answers are grouped together for ease of use. The sources are searched on a regular basis, and the tables on the webpage are updated accordingly.

## Results

We synthesized the guidelines and produced both a short practical checklist (Textbox 1) for clinicians to consider before, during, and after the consultation, and detailed textboxes (Textboxes 2-8) with a full summary of all the guidelines. Different guidelines use different terms (for example, telemedicine, telepsychiatry, videoconferencing, and telephone consultation). In our textboxes we have used the original terms from each guideline, and a glossary of definitions is included at the beginning of Textbox 2. Textboxes 2-8 cover general guidance (for all ages) with specific-labelled sections for children and adolescents, and older adults. The most up to date and detailed versions of the textboxes are available online [15].

**Checklist of things to consider before, during, and after the telepsychiatry consultation (detailed guidance for each point is contained in the numbered sections of the other textboxes).**

**Before the consultation**

- *Consult relevant national guidance for your country* (*section 2a*, in Textbox 3)
- *Consider information governance issues* and the *information technology* (*IT*) *system* that you and your patient will be using (*section 2b*, in Textbox 3)
- *Prepare the patient*: ensure the patient has relevant information before the consultation (*section 3a*, in Textbox 4)
- *Prepare yourself*:
  - Be familiar with the IT system you will use (*section 3b*, in Textbox 4)
  - Ensure your environment is set up appropriately (*section 3b*, in Textbox 4)

**During the consultation**

- Starting the consultation: *use a written checklist* such as the one shown below, derived from the American Psychiatric Association’s Telepsychiatry Toolkit [16] (*section 4a*, in Textbox 5):
  - *Name of clinician and patient* (eg, “Hello, I am Dr AB. Am I speaking to Mrs CD? Is there anyone else in the room you want me to be aware of?”)
  - *Location of the patient* (eg, “Can you let me know where you are right now? It is important for me to know this before each session.”)
  - *Immediate contact information for clinician and patient* (eg, “If we get cut off for any reason, how else can I reach you? If there is an emergency, you can also reach me at...”)
  - *Expectations about contact between sessions* (eg, “Although we are connecting in real time here and now, I want to review how we will communicate outside of these video visits. [Insert plan and note you cannot respond in real time outside of these visits.]”)
  - *Emergency management plan between sessions* (eg, “Should an emergency happen between visits, the plan that we have made is for you to [insert plan].”)
- Alternative checklists are available at [17,18]
- During the consultation focus on: (*section 4b*, in Textbox 5)
  - *Communication* (include nonverbal communication, slow down your speech, allow pauses, look at the camera)
  - *Contingencies/backup plan* in case of difficulties (IT or clinical issues), with contact details confirmed
  - *Confidentiality* (confirm you are talking to the correct person and they are expecting a mental health assessment, who else is in each room, manage your own environment)
  - *Consent* (discuss the possible limitations of remote contact and security of the IT platform)
  - *Confidence* (in using technology, give a clear plan if IT fails and clear plan at the end for the next steps)
- *Physical examination* is possible but *may need to be adapted* (*section 4c*, in Textbox 5).
- Consider *combining with other digital technologies* (eg, apps, websites for information, platforms for recording data such as mood symptoms; *section 4d*, in Textbox 5)
- Consider *safety and emergency plans* (*section 4e*, in Textbox 5)

**After the consultation**

- *Document appropriately*, just as you would for face-to-face contact with additional details relevant to telepsychiatry (*section 5a*, in Textbox 6)
- *Are the any special considerations?* (eg, older adults, child/adolescent, cultural issues, assessments by more than one member of the team; *section 6a-d*, in Textbox 7).
- *Are there any training issues to consider?* (*section 7a*, in Textbox 8)

**Background to telepsychiatry and what we know already (section 1).**

**1a. Definitions of terms relevant to telepsychiatry**

- *Telehealth* is the delivery of health care from a distance using technologies such as telephone, email, computer, interactive video, digital imaging, and health care monitoring devices. It is a broad term that covers many different types of health care including not only clinical but also nonclinical medical services such as education, research, and administrative functions.
- *Telemedicine* is a subset of telehealth. It includes many medical subspecialties (eg, telepediatrics, telepsychiatry, teleradiology, and telecardiology). It describes the use of technology to provide clinical medical services when the health care provider and patient are separated by a geographic distance.
- *Telepsychiatry* is a subspecialty of telemedicine and includes psychiatric assessments or follow-up interviews conducted using telephone calls, and audio and video digital platforms (videoconferencing, video consultation).

**1b. Is telepsychiatry a new skill and what do we know about it?**

- Videoconferencing in psychiatry began during the 1950s. By the 2000s, it was accepted as effective but slightly different from in-person care, and research in outcome studies provided a platform for *practice guidelines* (eg, the American Telemedicine Association in the United States). It has been applied successfully to *many cultures and international settings*. Telepsychiatry is equivalent to in-person care in *diagnostic accuracy, treatment effectiveness, and patient satisfaction. Patient privacy and confidentiality issues parallel in-person care*. It uses *specialty expertise* effectively, facilitating patient-centered and integrated care.

**1c. What is the evidence supporting telepsychiatry?**

- Good outcomes depend on *high-quality clinicians, organizations* (including leadership, clinical, technical, and administrative teamwork), and *technology*.
- The evidence base is substantial, and outcomes have been measured as follows [19]:
  - *Feasibility* rating: outstanding (based on satisfaction and usability)
  - *Validity* rating: outstanding
  - *Reliability* rating: outstanding
  - *Satisfaction* rating: outstanding among patients, psychiatrists, and other professionals
  - *Cost and cost-effectiveness* rating: similar to in-person or better
  - *Clinical measures*:
    - Interviewing, assessment, cognitive testing, and others: outstanding
    - Disorders include depression, anxiety, psychosis, substance misuse, cognitive/attentional/behavioral (assistance for those with learning disabilities or dementia), personality/behavioral, and many others: outstanding
    - Settings well-studied include outpatient, primary care/medical: outstanding. Settings less well-studied include accident and emergency departments (A and E), prisons, in-patient units, and schools: similar to in-person care.

**1d. Are there any settings where telepsychiatry might be better than in-person care?**

- For *children and adolescents on the autistic spectrum, and adults with disabling anxiety it may be preferable*.
- A growing body of evidence suggests that telepsychiatry may have *significant added value in A and E***,** where it can improve liaison with outpatient mental health services and reduce transportation costs, in-patient and A and E use, and overall hospital costs. It can also improve care *within primary care settings and specialty care clinics, prisons, and nursing homes*.
- Previous work (before the coronavirus disease [COVID-19] pandemic) has described effective strategies for using telemedicine in *disasters and public health emergencies*.
- COVID-19 specific preliminary advice: consider using telemedicine as a strategy for *health care surge control using “forward triage”* to sort patients before they arrive at the hospital and reduce the number who need to be seen in person. *Respiratory symptoms* (as an indicator of early COVID-19) can be evaluated by telemedicine along with detailed *travel and exposure histories*. *Automated screening algorithms* can be built in with local epidemiological information to standardize screening. For example, more than 50 US health systems already have such programs, which could be adopted for use during the current pandemic.

**1e. What treatment modalities can I use in telepsychiatry?**

- Telepsychiatric interventions have demonstrated clinical utility with a variety of treatment modalities *including group, individual, and family therapies*. Evidence-based psychological treatments have yielded positive outcomes (eg, *cognitive behavioral therapy*, *interpersonal therapy*, *exposure therapy*, *psychodynamic psychotherapy*, and *dialectical behavioral treatment*), and *evidence-based pharmacological interventions* can be prescribed electronically after telepsychiatric assessments.

**Guidelines and information governance on telemedicine and telepsychiatry (section 2).**

**2a. Are there guidelines I should be aware of?**

- *UK guidance*
  - *Royal College of Psychiatrists (RCPsych; coronavirus disease [COVID-19] guideline)*:
    - Remote consultations should be *encouraged where safe and appropriate*.
    - Ideally, an *adjunct* to, rather than substitute for, face-to-face consultation
    - For *initial consultations*, this may be even more challenging but should go ahead where possible.
    - Show *sensitivity* to the patient’s comfort level with technology
    - Do not disadvantage *those with lack of digital literacy or no access to digital platforms*
    - *Use of telephone consultations may be sufficient* for lower risk conversations or to ensure engagement with those who lack digital technology or skills.
  - *RCPsych, private and independent practice special interest group of the RCPsych (PIPSIG), incorporating General Medical Council (GMC) guidance (pre–COVID-19 guideline)*:
    - The *standards expected of doctors* by the GMC also apply to digital consultation settings.
    - Consideration should be given to *any potential limitations*: GMC guidance is that a doctor must be able to undertake an adequate assessment, establish dialogue, and obtain consent including consent to the remote consultation process.
    - Consider the *security of the system used* (see section 2b)
    - *Legal issues*:
      - Consider the *limitations* of telepsychiatry
      - The GMC *does not permit disclaimers* regarding the quality of a consultation.
      - You *may not be indemnified* for patients who are not in the United Kingdom.
    - *General areas to consider:*
      - Remote video consultation *may not be suitable for everyone*.
      - *When telepsychiatry would be used* (eg, should the first consultation be face-to-face)
      - How will you assess *suitability of the client* for telepsychiatric consultation?
      - How will you assess *suitability of the equipment*?
      - How often suitability would be *reassessed*
      - Consider *patient safety*: discuss and agree on supplying the contact information of a family or community member if needed
      - Whether you are *indemnified*
      - *Confidentiality issues*
      - The *right of the patient to withdraw* at any time
      - The taking and storage of *clinical notes and correspondence*
  - *General Medical Council (GMC) (pre-COVID-19 guideline):*
    - Consent and continuity of care are key issues to remember when you are advising or prescribing treatment for a patient via remote consultation.
    - *Consent*
      - *Explicit consent should be sought*: include the *right to withdraw* at any time; if the consultation is *recorded, consent is essential* and a GMC requirement; give patients *information about all the options* available to them; tailor the information you give and check that they have understood it. If you are not sure, consider whether it is safe to provide treatment and whether you have valid consent. You must ensure you can *assess a patient’s capacity*. If a patient lacks capacity to make a decision, consider whether remote consultation is appropriate.
    - *Continuity of care*
      - Ask the patient for *consent to get information and a history from their general practitioner* and to send details of any treatment plan. If the patient refuses, explore their reasons and explain the potential impact of their decision on their continuing care. If the patient continues to refuse, consider whether it is safe to provide treatment and make a record of your decision.
    - *If you are providing services remotely, remember to*:
      - Follow GMC guidance on consent and good practice in prescribing, work within your competence, check that you have adequate indemnity cover, discuss with your responsible officer at appraisal.
    - *Face-to-face treatment* may be preferable when there are complex needs or higher risk, you do not have access to the patient’s medical records, you do not have a safe system in place to prescribe, you need to complete a physical examination, you cannot give the patient all the information, or you are unsure about the patient’s capacity.
  - *National Institute for Health and Care Excellence (COVID-19 guideline)*:
    - Minimize face-to-face contact by offering telephone/video consultations, reducing nonessential face-to-face follow-up, and using electronic prescriptions and different methods to deliver medicines to patients.
  - *National Health Service (NHS) England (COVID-19 guideline):*
    - *Clinical teams should discuss with patients and families/carers in advance* about suitability and willingness to engage via technology.
    - Providers *may consider stratifying patients* where there is the highest risk of losing contact and agreeing how contact will be retained.
    - Where patients and carers live at a significant distance or are in isolation, *it may be appropriate to offer access to “virtual (ward) rounds”* (where a number of clinicians together consult remotely with a patient).
  - *UK guidance on remote prescribing:*
    - *Follow GMC guidance* on prescribing (outlined above)
    - *Follow UK legislation* on prescribing [20]
    - *Follow local guidance* for remote prescribing (see [21] for an example)
    - *Additional prescription requirements* may be required
    - Consider other *licensing restrictions*
- *US guidance*
  - *Federation of State Medical Boards (pre–COVID-19 guidelines*):
    - 49 state boards (plus the medical boards of the District of Columbia, Puerto Rico, and the Virgin Islands) require physicians engaged in telemedicine to be *licensed in the state in which the patient is located*.
    - 12 state boards *issue a special purpose license, telemedicine license/certificate, or license to practice medicine across state lines* to allow telemedicine. 6 state boards *require physicians to register if they wish to practice across state lines*.
    - Payment arrangements vary across states for telemedicine.
    - [22] summarizes US legislation related to telemedicine in different states.
  - *Centers for Medicare and Medicaid Services* recently broadened access to Medicare telehealth services in the context of COVID-19 on a *temporary and emergency basis.* Medicare can now pay for visits via telehealth across the country, including in patient’s homes, provided by doctors, nurses, clinical psychologists, and social workers. Prior to this, Medicare could only pay for telehealth on a limited basis (eg, in a designated rural area).
  - *American Psychiatric Association (Telepsychiatry)* does not give specific guidance but provides a practical “toolkit” of advice for general methods in telepsychiatry (not COVID-19 specific; sections are also referenced in relevant sections of this paper).
  - *Centers for Disease Control and Prevention (COVID-19 guideline*):
    - Explore alternatives to face-to-face triage and visits.
    - For example, use *available advice lines, patient portals, and online self-assessment tools*; identify staff to *telephone patients*; develop protocols so that staff can triage quickly; and have *algorithms to identify which patients can be managed by telephone* and which will need to be assessed in person.
    - Those with *respiratory symptoms must call before they leave home*, so staff can be prepared when they arrive.
  - American College of Physicians has produced an online course (open-access without certificate) on the use of telemedicine in general.
- *Singapore guidance*
  - *Singapore Medical Association (COVID-19 guideline*):
    - Assess the patient’s profile for *suitability*
    - Explain the *limitations* of telemedicine before consent
    - Recognize the *challenges and limitations*
    - Verify *patient identity* before proceeding and include in clinical documentation
    - Take a *thorough and comprehensive history*
    - Be confident that physical examination of the patient is unlikely to add critical information
    - Be aware of the *clinical “red flags,”* which would trigger the need for urgent in-person consultation, such as increased acute risk to self or others
    - *Clinical documentation* should be maintained at the same standard as an in-person consult.

**2b. What information governance issues should I consider?**

- *NHSX* has published *pragmatic guidance* on information governance since the outbreak of the COVID-19 pandemic, encouraging the use of videoconferencing to carry out consultations with patients and service users. The guidance states that it is acceptable to use tools such as Skype, WhatsApp, and Facetime as well as commercial products designed specifically for this purpose. The *consent of the patient is implied* by engaging in the consultation and clinicians should *safeguard personal/confidential patient information*.
- *Public Health England* strongly advise the use of remote access of NHS and essential services for anyone *over 70, with an underlying health condition, or who is pregnant* during the COVID-19 pandemic.
- RCPsych and PIPSIG suggest also considering:
  - Check that the *application is suitable* for a confidential psychiatric interview
  - Use a *secure system*
  - Have a *dedicated clinical account*
  - Make sure both parties have the *necessary technology*
  - Make sure both parties have the *skill to use the system*
  - Ask if an *advocate or carer* is present
  - Take *contact details* early in the proceedings
  - Agree who will contact whom in the event of a lost connection
  - Consider the *environment* beyond your video camera
  - Is there *anyone else* in the room with the clinician who cannot be seen? If so, introduce them and explain.
  - Does the *patient have anyone else* present in the room? Ask them to introduce themselves and move in front of the camera.
  - Consider the *volume* of loudspeakers and suggest that the patient does the same
  - Consider the use of *headphones*
  - Ask your *local information technology* (*IT*) *training/support team* for help (refer to [23])
  - Use a broadband internet connection of *at least 5 MB* upload/download
  - Choose a software solution that is compliant with your *local and national guidance*
  - Use a *secure, trusted platform*
  - Make sure your audio and video transmission is *encrypted* (follow local and national guidance)
  - Make sure your device uses *security features* such as passphrases and two-factor authentication
  - Use the latest *security patches and updates*; IT staff should approve of and manage your device.

**Tasks before the consultation (section 3).**

**3a. What should the patient know before the consultation?**

- Ensure the patient has *access to the technology* they require, including internet access, as well as the *skills to use it*. (If an administrator is setting up the call this could be something they can check or test as a trial run in advance.)
- Consider any problems with *accessibility*
- Do they have a *carer* who can facilitate the video consultation?
- Consider skills on a *case-by-case basis*

**3b. What should I do to prepare in advance?**

- Specific guidance is available through your *internal website or information technology training team*
- *Familiarize* yourself with the video consultation platform available
- *Test* the use of the platform and its features with a *colleague*
- *Make a note* of the features you might want to use and have a *summary sheet*
- *Restart* your computer every day
- *Close* any unnecessary programs and applications
- *Install recommended updates* from sources you trust
- Locate the volume control and *adjust the volume* or mute/unmute your speakers
- Use a *wired network connection* instead of Wi-Fi
- Sit a *comfortable distance* from the camera so your patient can see and hear you clearly
- Sit without windows or bright lights behind you
- Place your device on a *table or desk* facing you
- Keep *background noise* to a minimum

**During the consultation (Section 4).**

**4a. How should I start the consultation?**

- At the beginning of a video session with a patient, verify and document essential information and use a *checklist* (see Textbox 1).

**4b. What should I try to do throughout the consultation?**

- *Communication*:
  - Allow *nonverbal communication*: include your head, neck, upper body, and arms in the video screen
  - *Slow* your rate of speech and *pause longer* between sentences
  - Use *clear language*
  - *Look at the camera,* not at the patient’s eyes
  - Use any *features* such as a shared “white board” function you are familiar with
  - *Lighting and background* are important—plain, darker static/uncluttered background with light directly on your face may help.
  - Take *more time* over the introduction and *signpost* what is going to happen next.
  - Adjust your *position* before you start
  - *Avoid* looking away
  - *Give ample time* for a patient to hear your question and to reply
- *Contingencies*:
  - Have a clear understanding of what to do when the consultation is not going well
  - Have a *backup plan* for managing any technical difficulties and provide this via email
  - Check that you have the right mobile *telephone number* and agree who will contact whom in the event of a lost connection
  - Brief the patient that if you do not feel able to complete an adequate assessment you will discuss what steps to take next
  - Ideally have this process *mapped out in front of you* until you are familiar with it
  - *Practice the “script”* that you might want to use for managing contingencies
  - Make sure the technology is *charged or plugged* in and have a backup device available
- *Confidentiality*
  - If the patient is new to you, verify *they are the right person* and that they are expecting to review *their mental health*
  - Check *who is in the room* with the patient
  - If the patient is in *a public place*, consider with them whether it is appropriate to continue
  - *Manage your own environment* and avoid sensitive, personal details in the background
  - *Blur the background behind you* if possible (or use a standard, preset background) to hide personal details and present a professional appearance
  - Use a *dedicated clinical account*
- *Consent*
  - Be clear about the *limitations* of the assessment or review and ask whether they have any concerns
  - Ensure that you are clear about the security of the platform you are using, that it is fit for the purpose and be able to discuss this
  - Discuss with the patient about recording the session, agree what might be useful and only for private use
- *Confidence*
  - Be confident in *using the technology* and have a *clear plan* of what to do if something goes wrong
  - Say if it is not possible to do a good enough review and develop a clear plan of what to do next

**4c. How do I manage examinations that require physical interactions?**

- Although physical examination may be restricted, a significant amount of information can be obtained remotely**.** For example, a good representation of a neurological exam can be obtained [24]. Some aspects may also require a family or staff member to help.

**4d. How can I integrate telepsychiatry with other digital technologies?**

- Examples include health information websites, connecting with others through chat rooms or social media, using mental health mobile apps, email, or other technologies
- In general, *assess patients’ use of other technologies*: what they use, how often, and why they prefer certain types. How does it influence their life or affect their understanding of their presenting problem? How does it affect the therapeutic relationship? Are they aware of safety issues?
- Key considerations about *website health information, texting (SMS), and email*:
  - Health information on the internet for the public *is rarely regulated*.
  - Seek out information from organizations/institutions/businesses that have some *oversight/expertise*
  - Remember to *verify* the identification of the person on the other end of the receiving technology
  - Be cautious about *privacy/confidentiality* issues when using new digital communications that are not secure
  - Regulate requests for other *contact between visits* and use email/text only for patients who maintain in-person follow-up
- *Social media and professionalism*:
  - Be mindful of *privacy, professional image, confidentiality, and expectations*
  - Follow professional recommendations about *professionalism and social media*
  - *Consider pros/cons of gathering information about patients via search engines and social media*
  - For blogs, microblogs, and comments: *“pause before posting”* to consider the impact
  - *Separate* personal and professional life
- Apps (for mental health and general well-being) *and other digital technologies*:
  - Mobile health apps have many potential advantages. They are easily *accessible*, have increasing *precision and therapeutic potential, and offer unique insights into physical and cognitive behavior*. However, they are developed and shared at a fast rate, so it is hard to assess *clinical efficacy, safety, and security*, and depends on the user so may not be as effective in clinical settings as in research**.**
- *Regulatory bodies for apps and digital technologies:*
  - *The US Food and Drug Administration regulates mobile medical apps* [25] but prioritizes monitoring and approval of mobile apps that directly control medical devices or function as these, which excludes most mental health–related resources from evaluation. A pilot in 2019 to “pre-certify” digital health developers who have already shown credibility/excellence in software design speeds up the process but may introduce bias. They are now piloting a program that accredits developers and software companies, not the technology itself.
  - The *National Health Service* (*NHS*) *Apps Library* [26] contains recommended digital health tools but does not regulate development or enforce data security standards and offers advice (rather than regulation) only.
  - The *NHS also collaborated with National Institute of Health and Care Excellence* (*NICE*) to establish credentials for digital health tools or *“Digital Health Technologies”* [27]. NICE assesses the evidence base as well as its financial footprint.
- Evaluation websites
  - Often show a lack of concordance between ratings of the same apps, use qualitative measures, and are quickly out of date
  - The *American Psychiatric Association app evaluation framework* [28] suggests that patients and clinicians ask questions across four areas, in order of descending importance: safety and privacy, evidence, ease of use, and interoperability [29]. This could also be supplemented with a self-certification checklist completed by developers or volunteers as a public, interactive approach.

**4e. What about safety and emergency considerations?**

- When evaluating patient safety, assess the level of *agitation*, the potential for *harm to self or others*, as well as any *safety hazards*. Be familiar with *where* the patient is located, including any *immediate staff* who will be available for emergency procedures, and ways to obtain *collateral information*. Technology can be used to manipulate the image and sound quality of the video to assess agitation or patient safety (see also section 6b in Textbox 7, for further guidance).

**What should I do after the consultation (section 5)?**

**5a. What do I need to document during and after the assessment?**

- Clinical documentation as usual, with the addition of the *time, date, remote site location, time spent with the patient, the location and personnel*, as well as the *full clinical history, mental state examination, diagnosis, and treatment plan that you would normally document*

**What about subspecialties and special situations (section 6)?**

**6a. Are there any special considerations for older adults?**

- *Positive outcomes have been described for* satisfaction, validity/reliability, and clinical outcomes, and *satisfaction has been superior* for patients, families, carers, and providers. There has been effective use in the treatment of *depression, anxiety, dementia/cognitive impairment, and associated behavioral problems*.
- Clinicians need to make a few key changes: it is helpful to have *previsit accounting* of general events and the patient’s attitude, comments, complaints, sources of information, and clinician observations, and the clinical examination *may require staff or family assistance* (see section 4c in Textbox 5).
- The benefits are that *family and carers are appreciative of services, assessment, and cognitive intervention, and outcome results are similar to in-person care*. In addition, the clinician is part of an *interdisciplinary team* who can all be easily and efficiently connected through telemedicine and deliver specialty expertise for *nursing home and home outreach.*

**6b. What about child and adolescent patient consultations?**

- *Evidence base:*
  - Telepsychiatry services have been successfully used with diverse populations *across diagnoses (eg, depression/attention-deficit/hyperactivity disorder/tics/obsessive compulsive disorder/autism/psychosis) and settings (including urban/rural, community/school/home/inpatient/forensic [juvenile justice])*. Multiple studies have shown its *feasibility*. Referrers, psychiatrists, and families report *high satisfaction* with telepsychiatry services. The ability to establish a *therapeutic rapport* with youth and families through telepsychiatry is well established.
- *Practical suggestions in telepsychiatry with young people*
  - *Comment on real features* in the patient’s room so they know you can see and hear them.
  - *Greet patients*: How are you? Can you see and hear me OK?
  - *Replace the handshake* (eg, with a wave or fist bump)
  - *Use nonverbal communication*: facial expression, gestures, eye contact, tone of voice
  - *Ask about physical comfort*: privacy, temperature, lighting
  - *Adjust your voice*: slower and clearer, with longer pauses after questions to avoid talking over each other.
  - *Nod and smile frequently*
  - *Maintain eye contact* (look at the camera)
  - *Direct the family arrangement* at the beginning so all remain visible throughout the consultation and make sure the lighting is adequate
  - Use the *zoom and wide function* if needed
  - Position yourself so your *eyes appear one-third of the way down from the top of the screen*
  - Arrange the patient’s picture on your screen *as close as possible to your camera* (to allow for “relative eye contact”)
  - *Keep both cameras still*
  - *Ensure adequate lighting*
- *Safety*
  - Establish at each site what the *infrastructure and emergency management protocols* are in place, which can be adapted
  - Ensure that the *contact information* for parent(s)/caregiver(s) are up to date and available
  - In *hospital and community settings* these will be well established, but in *nontraditional settings* (eg, shelters for families and children), these will need to be developed and established before starting.
  - Emergency management is a *team effort*: *identify on-site staff* who can help by physically intervening during the emergency. Community resources must be identified to incorporate into *emergency management protocols and the patient’s care plan*.
  - Safety and mobilization procedures at a patient site should be both *accessible to staff for review and an integral part of their training*.
  - *Manipulate the technology* to maximize video and audio quality to assess signs of agitation, substance use, and medication side effects.
  - If technology falters, have *a preplanned backup emergency management plan* (eg, calling a named coordinator at site to enter room and ensure safety)
- *Training*
  - Official guidelines for training competencies have not yet been established.
  - *Primary skill areas* (see section 7a in Textbox 8) all have special applications for children and adolescents.
  - Clinicians need to learn to *increase nonverbal communication by approximately 15-20%* for effective use on screen.
  - Be *collaborative* with all staff at patient site across disciplines to integrate into the team remotely
  - Be sensitive to *cultural and community issues*
  - *Help staff* to be comfortable with telepsychiatry
  - *Be flexible in your role* in the child’s system of care and vary your role depending on the resources available at the patient site
  - *Understand legal/policy/regulation guidelines*
- *Developing a therapeutic space and establishing rapport*
  - *Clinician’s room*:
    - *Minimize detail* (to facilitate the camera’s focus and not distract the patient)
    - *Do a room tour* to show privacy and welcome patient and family
    - *Show the therapist from waist up* (like a news broadcaster) to include all nonverbal communication
    - Include in frame *any tools or gadgets you intend to use*
  - *Patient’s room*:
    - *Large enough* for patient plus family plus any caregivers/staff attending
    - *Large enough* to assess patient’s physique, motor skills, behavior, mental status examination, gross motor and fine motor skills, affect, and rapport
    - For one participant, they should sit *2-4 feet* away from the camera. For each additional participant, *another 2 feet back* from the camera will keep all participants in the screen’s framing.
    - Camera needs to be *far enough* away that young children are always in shot, even if they move to play on the floor
    - Consider the *selection of toys*: useful as a distraction but avoid noisy toys and those with lots of pieces; ideal is a small table with paper and crayons
    - In general, *control use of electronics* by patients during the interview but be flexible (eg, for teens)
    - Use *creative ways to establish rapport* (eg, comment on the child’s toys/clothing/room features to show you can really see them, check they are happy with the room layout, ask them to check their own image on the screen)
    - Two-thirds of the meaning of a consultation comes from *nonverbal communication.* It is often not what is said, but how it is said, that matters most to our patients.
    - Be creative with *seating arrangements*: children can sit next to or between their parents, on their lap, or in front on a chair or on the floor.
    - If *a hyperactive or autistic child* cannot remain in the camera frame, keep the parent(s) in and call the child back to the camera when they need to answer a question.
    - If a child *refuses to sit* within the camera frame and behavior management strategies do not work, consider seating them further from the camera but in the frame, or allowing more privacy for part or all of the session.
- *School-based telepsychiatry*
  - There are *many advantages*: reduced travel time for psychiatrists, reduced parents’ work leave, reduced child absence from school, increased attendance at psychiatry appointments, facilitates a team-based approach with earlier interventions and better compliance. However, the following should be considered: finding a *private/secure space, understanding and respecting school staff, policies and structures, knowledge of existing school support and learning support, continuity outside school* (evenings, holidays, etc). Ideally, clinicians should use a *hybrid approach* with some in-person meetings at the beginning. Clinicians should identify which staff will support with practical arrangements and in the meetings if needed.
- *Forensic (juvenile justice) settings*
  - Can be *challenging*: some young people are reluctant to speak with psychiatric and other mental health staff, particularly if sessions interfere with their participation in recreational activities or there are concerns about staff being present and confidentiality.
  - Telepsychiatrists must *define their role* in the youth’s system of care and treatment (ie, to clarify a forensic vs direct care role).
  - *On-site therapists* (but not correctional staff) typically participate in sessions to aid the psychiatrist in obtaining pertinent patient information and to facilitate clinical care.
  - *Background information and reports* may be available in advance, and these should be used proactively in the interview.
  - Telepsychiatrists should be familiar with regulations regarding *consent to pharmacological treatment of minors in forensic settings*.
  - Telepsychiatrists may ask staff to provide a *“virtual tour”* of the facility with a mobile device to ensure privacy, security, management of mental health records, and other concerns.
- *Cultural issues*
  - See section 6c; note that *family structure* may differ in different cultures; take care to use *professional interpreters*, not family members
- *Behavior management*
  - Evidence-based behavior management training can be offered via telepsychiatry in clinic and home settings.
  - Psychiatrists can both model and coach parents on the concepts of behavior management in real time.
  - In clinic, staff can clarify subtleties in the child’s behavior that may not be evident through videoconferencing.
  - Treatment can be offered in naturalistic settings such as the home, potentially providing more ecologically valid assessments and interventions.
  - Develop a *safety and crisis plan* (including contact lists of trusted family/friends, local general practitioner, and emergency services) at the beginning in case the child’s behavior becomes unmanageable or unsafe during a session

**6c. How should we consider cultural issues?**

- Be *knowledgeable about the culture(s) and environments* in which care is provided
- Be aware that cultural differences can be highlighted by the patient and provider locations
- Assess how a patient’s cultural background influences their *comfort and use of technology*
- Consider how best to *adapt* their communication style and clinical processes

**6d. How do we manage a patient interaction when more than one member of the team is present on the call?**

- It is important to incorporate each member in the process. Each member of the team at both sites should *introduce* themselves with their name, title, and role and make sure that the patient understands the nature of the encounter. After interviewing and examining the patient, the clinician should *check in with each team member* for their input and to *clarify the diagnostic impression and feasibility of a treatment plan*.

**Training and service needs (section 7).**

**7a. How can I prepare to be a good telepsychiatrist?**

- *Useful previous experience* includes settings with communication skills and adjustments for the setting, audience, and objectives of the event (eg, public speaking, acting, coaching, and media experience)
- *General considerations*
  - *Practice and self-observe* (for example, by recording a practice interview with a colleague)
  - Focus on *patient-centered, respectful, active listening, expressing empathy, cultural sensitivity, use of nonverbal behavior and replace physical contact with welcoming statements*
  - In team assessments, remember *introductions, engaging others* to get involved, and *giving directions or ground rules* to provide *structure*
  - Use elements of *good public speaking*
  - *Plan* and manage the session, be *organized*, consider an *opening script* for new assessments
- Clinical considerations
  - Maintain the *standard of care and quality* of service
  - Document *informed consent*
  - *Engage* the patient and put them at ease
  - *Previsit preparation* is helpful
  - *Allocate enough time*: video interviewing takes longer and requires more concentration
  - *The setting/room*: both ends private/secure, announce anyone who is unseen to the patient, check lighting, and check equipment
  - Check in with the client at the *end of the session*
  - *Minimize interruptions* and reduce the amount of information dispensed
  - *Dress appropriately* and *project your voice and other gestures about 15% greater* than in-person
  - *Adjust to age* (eg, toys and table for kids; support person for older adults)
  - *Adapt your clinical examination* where needed
  - *Encourage family members* to attend if possible and the patient agrees

We used a synthesis of the guidelines to answer specific questions generated by clinicians on telepsychiatry and related digital technologies using the following sources: *American Psychiatric Association (APA), CDC, CMS, FSMB, General Medical Council (GMC), Massachusetts General Hospital Department of Psychiatry, NHS Wales, NHSX (a joint team from the Department of Health and Social Care and NHS England and NHS Improvement), the National Institute for Health and Care Excellence (NICE), NMC, Public Health England, Royal College of Psychiatrists,* and *Singapore Psychiatric Association.*

The full list of the sources searched, specific sources used, and further detail with webpage links on each point are contained in Multimedia Appendix 1.

## Discussion

In this paper we have summarized the available evidence base for guidelines in telepsychiatry across a wide variety of treatment modalities and populations. Telepsychiatric treatment has not previously been implemented on the scale and speed demanded by the current COVID-19 crisis. This is, at least in part, because mental health clinicians feel a lack of detailed knowledge and experience in telepsychiatry, and often express concerns about establishing rapport and therapeutic alliance [12], and key areas such as assessing risk and safeguarding [7]. Developing skills in telepsychiatry, including competence in creating a so-called “webside manner” [30] requires knowledge, training, and experience. Building confidence and knowledge is the first important step and requires easy access to a reliable source of existing information, such as that summarized in our synthesis of guidance.

Training clinicians is the next key area, both in the practical use of remote consultations and in the use of digital interventions, treatments, and mobile apps. Frameworks for training exist [31,32] but have not been widely implemented in practice. However, teaching experience such as with psychiatry residents has been positive, and there are already many successful examples of teaching telemedicine [10]. Training takes time, so it does not offer an immediate solution to the current crisis, but it will build capacity for increased access to care for the mental health sequelae of the current crisis and help to prepare for the next.

Training for clinicians is only one part of the story. It is also important to ensure that all patients have access to telepsychiatry. Many people may be prevented from accessing digital health, either because of a lack of skill or competence, or because of a lack of suitable access to reliable internet connections, smartphones, or similar platforms [10]. These reasons affect particularly the most vulnerable in society; issues such as age, language, cultural background, and homelessness can all contribute. Training programs for patients [33] have been effective in increasing patients’ confidence and competence, but practical access to relevant technology is a wider societal issue. Clinicians also need to be aware that telepsychiatry is a tool for consultation, which needs to be adapted flexibly and creatively to each patient’s needs, preferences, and circumstances. As the guidelines in section 2a of Textbox 3 outline, a pragmatic approach needs to be taken, particularly during the constraints of the COVID-19 pandemic; for example, telephone consultations may be used to facilitate engagement with those who lack access to digital technology or the skills or confidence to use video platforms. Alternatively, a hybrid or blended approach (as discussed later) may be the best option for some patients after weighing the potential risks and benefits for an individual, even during the COVID-19–related constraints imposed on in-person contact. Careful preparation in advance, as outlined in section 3a of Textbox 4, can also help to ensure that time spent during the consultation is as comfortable and effective for the patient as possible. Part of this preparation should also be to remind patients that their usual rights (for example, for a second opinion, to make a complaint, or decline a treatment) are the same whether the meeting is in person or delivered remotely.

As well as clinicians and patients, organizations providing services in mental health care also need to implement changes to reflect the acceptance of telepsychiatry as a valid and beneficial treatment option. In the short-term response to the crisis, there have been examples of organizational changes such as the lifting of state and federal regulation in the United States that had previously been barriers [3]. Longer term, it is unclear whether these changes will return to pre–COVID-19 rules or not. To ensure that these changes continue, clinicians and organizations need to campaign proactively for long-term change [3]. Central to this will be widespread dissemination of evidence-based guidance to clinicians who can then gain the confidence and competence needed to implement telepsychiatry and to extend its use beyond the short-term crisis. Research on the implementation of telepsychiatry will also need to assess outcomes and their cost-effectiveness, to support long-term change to systems of payment and insurance [12].

One route to demonstrating the clinical efficacy and cost-effectiveness of telepsychiatry, particularly as a longer-term strategy, will be to assess its use not only as a replacement for face-to-face care but also as a possibility for an enhanced level of care. It offers options for patients to personalize their treatment in choosing how they want to see their psychiatrist. In the aftermath of COVID-19, many patients may choose to continue with remote consultation because of its advantages such as privacy (reducing stigma) and convenience. For a small minority, particularly those with psychosis or persecutory beliefs, remote psychiatry may seem less trustworthy than in-person care. Post–COVID-19, patients will be able to individualize their care, balancing the pros and cons for their own treatment, with many opting for a hybrid model across different remote and in-person settings. Clinicians will need to balance the strengths, limitations, and adjustments needed for each approach and feel competent in using all media [34].

Telepsychiatry also offers options for easily combining with other digital technologies. These include platforms for monitoring symptoms such as mood monitoring systems [35] or combining care with apps [34]. Smartphone apps offer advantages in accessibility, insights into physical and cognitive behavior, and a range of resources designed to aid health. However, there are challenges to be addressed; clinicians and patients need to assess these digital resources for efficacy, safety, and security so that only high quality and clinically effective apps are offered to patients [36]. Although there are app comparison sites (for example, [37]), the volume of work in appraising apps is ever increasing, so sites often show a lack of concordance between ratings of the same app, may use qualitative measures, and are quickly out of date. For example, the initial version of the NHS Apps Library [26] was withdrawn after criticism of a lack of consistency in evidence of effectiveness, privacy, and confidentiality. The library was relaunched but now offers advice rather than regulation. The APA app evaluation framework [29] suggests that users (patients and clinicians) ask questions across the areas of safety and privacy, evidence, ease of use, and interoperability, supplemented if possible with a self-certification checklist completed by developers or volunteers on a frequent basis. Ideally, this would mean that a patient could filter categories for app choices that meet their requirements across the areas assessed. Apps are twice as effective when used with a clinician [38], so their combination with telepsychiatry presents exciting opportunities. Similarly, the addition of apps to provide lifestyle interventions [10] is particularly relevant during the current restrictions and will be an important opportunity both during the acute crisis of COVID-19 and afterwards.

Some potential limitations of our work should be acknowledged. The original search was restricted to English language sources, but international collaborators are producing translations (for example, in French, Turkish, and Chinese) and adaptations for local use before dissemination. Any summary of guidance needs to reflect the global perspective, as implementation and advice on telemedicine and telepsychiatry vary. Balancing guidelines across countries is a key area of concern when producing a summary of evidence. UK guidelines on telepsychiatry (for example, those from the Royal College of Psychiatrists and GMC) are generally more cautious and emphasize possible constraints, whereas US views are more expansive, with stronger emphasis on the evidence base and the potential benefits of combining with apps and other technologies. Although valuable COVID-19 specific mental health treatment guidance is starting to be generated (for example, in the UK, COVID-19–specific guidance in how to remotely deliver the NICE recommended cognitive therapies for PTSD, social anxiety disorder, and panic disorder [39]), the vast majority of guidance and evidence on use of telepsychiatry is based on the pre–COVID-19 era. Although there is some preliminary data on the use of telepsychiatry in other disasters [40], the COVID-19 crisis is different to previous pandemics. Thus, this synthesis of guidance contains a combination of pre–COVID-19 guidelines mixed with recent crisis guidelines. Although the COVID-19 guidelines are more pragmatic and perhaps more relevant, this combination does produce inconsistencies (for example, on whether consent to telepsychiatry meetings is implicit or needs to be explicit). 

Most of the guidelines focus on practical implementation in general adult psychiatry (with some specific guidelines for younger and older patients). Although this is helpful, if telepsychiatry is to be integrated into routine care in the longer term, then guidance also needs to focus on those areas and patient populations who may be more difficult to engage, for example, those with learning disabilities, psychotic symptoms including persecutory beliefs, personality disorders, and eating disorders. In addition, the guidelines do not clearly differentiate between initial assessment and follow-up. Although the general principles may be the same, the focus of these different encounters may vary, and these are not clearly defined. The existing guidelines do not specifically include the concept of hybrid or blended care, which is the route most likely to be effective in the longer term. This involves the combination of telepsychiatry with not only in-person care but also other digital technologies. Frameworks for telepsychiatry alone are well established and are more preliminary for digital technologies, and there is no clear guidance on the combination of all. Hybrid care will be key in the way forward post–COVID-19 to allow clinicians and their patients to choose the individual combination of care that offers the most advantages. To do this, mental health clinicians need to become adept at managing hybrid clinician-patient relationships and have a detailed understanding of the advantages and limitations of all the tools they use to care for patients [34].

The acute COVID-19 pandemic and its aftermath present new challenges. Although the benefits of implementing telepsychiatry are clear, this can only be fully realized if clinicians see this as an opportunity for not only a short-term solution to a crisis but also a cultural shift and opportunity to integrate the benefits of telepsychiatry into a model of blended care in the future. The acute crisis can be an opportunity to develop and implement digital mental health in a collaborative environment between patients, carers, and clinicians. This is an important moment where mental health professionals and health care providers can embrace telepsychiatry and introduce new and innovative strategies to make long-lasting improvements in the access to and quality of mental health care provision.

## Appendix

Multimedia Appendix 1 Supplementary material.

## Abbreviations

APA: American Psyciatric Association
CDC: Centers for Disease Control and Prevention
CMS: Centers for Medicare and Medicaid Services
COVID-19: coronavirus disease
FSMB: Federation of State Medical Boards
GMC: General Medical Council
NHS: National Health Service
NICE: National Institute of Health and Care Excellence
NIHR: National Institute for Health Research
NMC: Nursing and Midwifery Council
PTSD: posttraumatic stress disorder
SARS-CoV-2: severe acute respiratory syndrome coronavirus 2

## References

[1] Fauci AS, Lane HC, Redfield RR (2020). Covid-19 — navigating the uncharted. N Engl J Med.

[2] Gates B (2020). Responding to Covid-19 — a once-in-a-century pandemic?. N Engl J Med.

[3] Shore JH, Schneck CD, Mishkind MC (2020). Telepsychiatry and the coronavirus disease 2019 pandemic-current and future outcomes of the rapid virtualization of psychiatric care. JAMA Psychiatry.

[4] Rogers JP, Chesney E, Oliver D, Pollak TA, McGuire P, Fusar-Poli P, Zandi MS, Lewis G, David AS (2020). Psychiatric and neuropsychiatric presentations associated with severe coronavirus infections: a systematic review and meta-analysis with comparison to the COVID-19 pandemic. Lancet Psychiatry.

[5] Brooks SK, Webster RK, Smith LE, Woodland L, Wessely S, Greenberg N, Rubin GJ (2020). The psychological impact of quarantine and how to reduce it: rapid review of the evidence. Lancet.

[6] Sucala M, Schnur JB, Constantino MJ, Miller SJ, Brackman EH, Montgomery GH (2012). The therapeutic relationship in e-therapy for mental health: a systematic review. J Med Internet Res.

[7] Cowan KE, McKean AJ, Gentry MT, Hilty DM (2019). Barriers to use of telepsychiatry: clinicians as gatekeepers. Mayo Clin Proc.

[8] Greenhalgh T, Wherton J, Shaw S, Morrison C (2020). Video consultations for covid-19. BMJ.

[9] FAQs. National Leadership and Innovation Agency for Healthcare.

[10] Torous J, Jän Myrick K, Rauseo-Ricupero N, Firth J (2020). Digital mental health and COVID-19: using technology today to accelerate the curve on access and quality tomorrow. JMIR Ment Health.

[11] Telepsychiatry toolkit. American Psychiatric Association.

[12] Torous J, Wykes T (2020). Opportunities from the coronavirus disease 2019 pandemic for transforming psychiatric care with telehealth. JAMA Psychiatry.

[13] Smith K, Ostinelli E, Cipriani A (2020). Covid-19 and mental health: a transformational opportunity to apply an evidence-based approach to clinical practice and research. Evid Based Ment Health.

[14] Welcome to the Oxford Precision Psychiatry Lab (OxPPL). NIHR Oxford Health Biomedical Research Centre.

[15] Digital technologies and telepsychiatry. NIHR Oxford Health Biomedical Research Centre.

[16] Standard operating procedures and protocols for telehealth. Digital Psych.

[17] Covid-19: remote consultations. NICE.

[18] Telemedicine: the essentials. The College of Family Physicians of Canada.

[19] Clinical outcomes. American Psychiatric Association.

[20] Prescription only medicines. Legislation.gov.uk.

[21] (2020). COVID-19 medicines information memo. NIHR Oxford Health Biomedical Research Centre.

[22] Telemedicine legislation. GovHawk.

[23] COVID-19 Information Governance advice for staff working in health and care organisations. NHSX.

[24] (2020). Free webinar: telemedicine and COVID-19 - American Academy of Neurology. YouTube.

[25] Digital health innovation action plan. U.S. Food and Drug Administration.

[26] NHS Apps Library. NHS.

[27] Evidence standards framework for digital health technologies. NICE.

[28] Mental health apps. American Psychiatric Association.

[29] App evaluation model. American Psychiatric Association.

[30] McConnochie KM (2019). Webside manner: a key to high-quality primary care telemedicine for all. Telemed J E Health.

[31] Hilty D, Chan S, Torous J, Luo J, Boland R (2020). A framework for competencies for the use of mobile technologies in psychiatry and medicine: scoping review. JMIR Mhealth Uhealth.

[32] Hilty D, Maheu MM, Drude KP, Hertlein KM (2018). The need to implement and evaluate telehealth competency frameworks to ensure quality care across behavioral health professions. Acad Psychiatry.

[33] Hoffman L, Wisniewski H, Hays R, Henson P, Vaidyam A, Hendel V, Keshavan M, Torous J (2020). Digital opportunities for outcomes in recovery services (DOORS): a pragmatic hands-on group approach toward increasing digital health and smartphone competencies, autonomy, relatedness, and alliance for those with serious mental Iilness. J Psychiatr Pract.

[34] Shore JH (2020). Managing virtual hybrid psychiatrist-patient relationships in a digital world. JAMA Psychiatry.

[35] Goodday SM, Atkinson L, Goodwin G, Saunders K, South M, Mackay C, Denis M, Hinds C, Attenburrow M, Davies J, Welch J, Stevens W, Mansfield K, Suvilehto J, Geddes J (2020). The true colours remote symptom monitoring system: a decade of evolution. J Med Internet Res.

[36] Leigh S, Flatt S (2015). App-based psychological interventions: friend or foe?. Evid Based Ment Health.

[37] PsyberGuide.

[38] Linardon J, Cuijpers P, Carlbring P, Messer M, Fuller-Tyszkiewicz M (2019). The efficacy of app-supported smartphone interventions for mental health problems: a meta-analysis of randomized controlled trials. World Psychiatry.

[39] COVID-19 resources. OxCADAT Resources.

[40] Reinhardt I, Gouzoulis-Mayfrank E, Zielasek J (2019). Use of telepsychiatry in emergency and crisis intervention: current evidence. Curr Psychiatry Rep.

