# Supplementary material for: COVID-19 and Telepsychiatry: Development of Evidence-Based Guidance for Clinicians
Source: JMIR Ment Health. 2020 Aug 28;7(8):e21108. doi: 10.2196/21108 (PMC7485934; doi:10.2196/21108)
Supplement: Multimedia Appendix 1 [file mental_v7i8e21108_app1.docx]

**Appendix**

**Sources searched:** Public Health England, Royal College of Psychiatrists (RCPsych), Royal College of Nursing (RCN), The National Association of Intensive Care and Low Secure Units (NAPICU), NICE, Royal College of Physicians, Healthcare Improvement Scotland, SLAM NHS Trust, NICE, NHS Wales, GMC, NHSX, NMC, CDC (Centers for Disease Control and Prevention), US Department of Labor, American Psychiatric Association, Massachusetts General Hospital Psychiatry, FSMB (Federation of State Medical Boards), CMS (Centers for Medicare & Medicaid Services), WHO, IASC (Inter Agency Standing Committee), UNICEF, WPA, Singapore Ministry of Health, Singapore Psychiatric Association, Singapore Medical Association, Health Canada (Government department), Canadian Psychiatric Association, Australian Government Department of Health, Royal Australian and New Zealand College of Psychiatrists.

**Sources used:** American Psychiatric Association (APA), Centers for Disease Control and Prevention (CDC), Centers for Medicare & Medicaid Services (CMS), Federation of State Medical Boards (FSMB), General Medical Council (GMC), Massachusetts General Hospital Department of Psychiatry, National Health Service Wales (NHS Wales), NHSX (a joint team from the Department of Health and Social Care and NHS England and NHS Improvement), The National Institute for Health and Care Excellence (NICE), Nursing and Midwifery Council (NMC), Public Health England, Royal College of Psychiatrists (RCPsych), Singapore Psychiatric Association.

**Supporting references and weblinks**

**1a Definitions of terms relevant to telepsychiatry**

<http://www.wales.nhs.uk/technologymls/english/faq1.html>

<https://www.rcpsych.ac.uk/about-us/responding-to-covid-19/responding-to-covid-19-guidance-for-clinicians/digital-covid-19-guidance-for-clinicians>

**1b. Is telepsychiatry a new skill and what do we know about it?**
<https://www.psychiatry.org/psychiatrists/practice/telepsychiatry/toolkit/history-of-telepsychiatry>

**1c. What is the evidence supporting telepsychiatry?**<https://www.psychiatry.org/psychiatrists/practice/telepsychiatry/toolkit/clinical-outcomes>

**1d. Are there any settings where telepsychiatry might be better than in person care?**<https://www.psychiatry.org/psychiatrists/practice/telepsychiatry/toolkit/clinical-outcomes>

<https://www.psychiatry.org/psychiatrists/practice/telepsychiatry/toolkit/return-on-investment>

<https://www.ehidc.org/sites/default/files/resources/files/Virtually%20Perfect%20-%20New%20England%20Journal%20of%20Medicine.pdf>

**1e. What treatment modalities can I use in telepsychiatry?**

<https://www.psychiatry.org/psychiatrists/practice/telepsychiatry/toolkit/clinical-and-therapeutic-treatment-modalities>

**2a. Are there guidelines I should be aware of?**

<https://www.rcpsych.ac.uk/about-us/responding-to-covid-19/responding-to-covid-19-guidance-for-clinicians/digital-covid-19-guidance-for-clinicians>

<https://www.rcpsych.ac.uk/docs/default-source/members/sigs/private-and-independent-practice-pipsig/pipsig-telepsychiatry-guidelines-revised-mar16.pdf?sfvrsn=30d4c605_2>

<https://www.gmc-uk.org/ethical-guidance/ethical-hub/remote-consultations>

<https://www.nice.org.uk/guidance/ng163/chapter/1-Communicating-with-patients-and-minimising-risk>

<https://www.nhsx.nhs.uk/covid-19-response/data-and-information-governance/information-governance/>

<https://www.nmc.org.uk/news/news-and-updates/how-we-will-continue-to-regulate-in-light-of-novel-coronavirus/>

<http://www.fsmb.org/siteassets/advocacy/key-issues/telemedicine_policies_by_state.pdf>

<https://track.govhawk.com/reports/2Nzd2/public>

<https://www.cms.gov/newsroom/fact-sheets/medicare-telemedicine-health-care-provider-fact-sheet>

<https://www.psychiatry.org/psychiatrists/practice/telepsychiatry>

<https://www.cdc.gov/coronavirus/2019-ncov/hcp/guidance-hcf.html>

<https://www.acponline.org/practice-resources/business-resources/health-information-technology/telehealth?utm_campaign=FY19-20_MD_TELEHEALTH_EML_CURRICULUM_MD9191_3A&utm_medium=email&utm_source=Eloqua&elqTrackId=59052d1d27704688a4d31f303180da66&elq=4857b1325cfe4ce5bd816bf95e3ed345&elqaid=5180&elqat=1&elqCampaignId=2289>

<https://www.sma.org.sg/UploadedImg/files/ncov2019/LeveragingTelemedicineInfectiousDiseaseOutbreak20200212.pdf> (page 4)

**2b. What information governance issues should I consider?**

<https://www.rcpsych.ac.uk/about-us/responding-to-covid-19/responding-to-covid-19-guidance-for-clinicians/digital-covid-19-guidance-for-clinicians>

<https://www.nhsx.nhs.uk/covid-19-response/data-and-information-governance/information-governance/covid-19-information-governance-advice-health-and-care-professionals/>

<https://www.rcpsych.ac.uk/docs/default-source/members/sigs/private-and-independent-practice-pipsig/pipsig-telepsychiatry-guidelines-revised-mar16.pdf?sfvrsn=30d4c605_2>

<https://www.psychiatry.org/psychiatrists/practice/telepsychiatry/toolkit/platform-software-requirements>

<https://www.psychiatry.org/psychiatrists/practice/telepsychiatry/toolkit/security-issues>

**3a. What should the patient know before the consultation?**

<https://www.rcpsych.ac.uk/about-us/responding-to-covid-19/responding-to-covid-19-guidance-for-clinicians/digital-covid-19-guidance-for-clinicians>

**3b. What should I do to prepare in advance?**

<https://www.rcpsych.ac.uk/about-us/responding-to-covid-19/responding-to-covid-19-guidance-for-clinicians/digital-covid-19-guidance-for-clinicians>

<http://www.mghtelehealth.org/patients/best-practices>

**4a. How should I start the consultation?**

<https://www.digitalpsych.org/uploads/1/2/9/7/129769697/session_start.pdf>

<https://www.bmj.com/content/368/bmj.m1182>

<https://www.cfp.ca/news/2020/03/26/3-26-1>

**4b. What should I try to do throughout the consultation?**

<https://www.rcpsych.ac.uk/about-us/responding-to-covid-19/responding-to-covid-19-guidance-for-clinicians/digital-covid-19-guidance-for-clinicians>

<https://www.psychiatry.org/psychiatrists/practice/telepsychiatry/toolkit/visual-nonverbal-aspects>

**4c. How do I manage examinations which require physical interactions?**

<https://www.youtube.com/watch?v=Pw-Jdy3-T9g>

**4d. How can I integrate telepsychiatry with other digital technologies?**

<https://www.psychiatry.org/psychiatrists/practice/telepsychiatry/toolkit/telepsychiatry-integration-with-other-technologies>

<https://www.jmir.org/2020/1/e15188/>

<https://bmcmedicine.biomedcentral.com/articles/10.1186/s12916-019-1447-x>

**4e. What about safety and emergency considerations?**

<https://www.psychiatry.org/psychiatrists/practice/telepsychiatry/toolkit/patient-safety-and-emergency-management>

**5a. What do I need to document during and after the assessment?**

<https://www.psychiatry.org/psychiatrists/practice/telepsychiatry/toolkit/clinical-documentation>

**6a. Are there any special considerations for Older Adults?**

<https://www.psychiatry.org/psychiatrists/practice/telepsychiatry/toolkit/geriatric-telepsychiatry>

**6b. What about Child and Adolescent patient consultations?**

<https://www.aacap.org/AACAP/Clinical_Practice_Center/Business_of_Practice/Telepsychiatry/Toolkit%20Videos/evidence_based.aspx>

<https://www.aacap.org/AACAP/Clinical_Practice_Center/Business_of_Practice/Telepsychiatry/Telepsych_Articles/Roth-Ramtekka-AACAP-News-web.pdf>

<https://www.aacap.org/AACAP/Clinical_Practice_Center/Business_of_Practice/Telepsychiatry/Telepsych_Articles/Roth-Ramtekka-AACAP-News-pt2-web.pdf>

<https://www.aacap.org/AACAP/Clinical_Practice_Center/Business_of_Practice/Telepsychiatry/Toolkit%20Videos/patient_safety.aspx>

<https://www.aacap.org/AACAP/Clinical_Practice_Center/Business_of_Practice/Telepsychiatry/Toolkit%20Videos/training.aspx>

<https://www.aacap.org/AACAP/Clinical_Practice_Center/Business_of_Practice/Telepsychiatry/Toolkit%20Videos/virtual_therapeutic_space.aspx>

<https://www.aacap.org/AACAP/Clinical_Practice_Center/Business_of_Practice/Telepsychiatry/Toolkit%20Videos/participant_arrangement.aspx>

<https://www.aacap.org/AACAP/Clinical_Practice_Center/Business_of_Practice/Telepsychiatry/Toolkit%20Videos/school_based.aspx>

<https://www.aacap.org/AACAP/Clinical_Practice_Center/Business_of_Practice/Telepsychiatry/Toolkit%20Videos/juvenile_justice.aspx>

<https://www.aacap.org/AACAP/Clinical_Practice_Center/Business_of_Practice/Telepsychiatry/Toolkit%20Videos/telepsychiatry_across_culture.aspx>

<https://www.aacap.org/AACAP/Clinical_Practice_Center/Business_of_Practice/Telepsychiatry/Toolkit%20Videos/behavior_management_training.aspx>

**6c. How should we consider cultural issues?**

<https://www.psychiatry.org/psychiatrists/practice/telepsychiatry/toolkit/use-of-telepsychiatry-in-cross-cultural-settings>

**6d. How do we manage a patient interaction when more than one member of the team is present on the call?**

<https://www.psychiatry.org/psychiatrists/practice/telepsychiatry/toolkit/team-based-integrated-care>

**7a. How can I prepare to be a good telepsychiatrist?**

<https://www.psychiatry.org/psychiatrists/practice/telepsychiatry/toolkit/learning-telemental-health>
